# ABO/Rh Blood Group and Cervical Cancer Survival: Results from Our Own and Other Studies

**DOI:** 10.7150/jca.95245

**Published:** 2024-07-09

**Authors:** Vincenzo Dario Mandato, Federica Torricelli, Valentina Mastrofilippo, Ilaria Vacca, Beatrice Melli, Matteo Generali, Gianluca Annunziata, Debora Pirillo, Giovanni D'Ippolito, Gino Ciarlini, Lorenzo Aguzzoli

**Affiliations:** 1 Unit of Obstetrics and Gynecologic Oncology, Azienda USL-IRCCS di Reggio Emilia, Reggio Emilia, Italy.; 2 Laboratory of Translational Research, Azienda USL-IRCCS di Reggio Emilia, 42122 Reggio Emilia, Italy.; 3 Molecular Pathology Unit, Azienda USL-IRCCS, 42123 Reggio Emilia, Italy.; 4 Clinical and Experimental Medicine PhD Program, University of Modena and Reggio Emilia, 41121 Modena, Italy.

**Keywords:** ABO blood group, Rh, cervical cancer, survival, age, hypertension

## Abstract

**Background:** Cervical cancer is the most common genital cancer worldwide and is mainly caused by a persistent human papillomavirus infection. Well-known prognostic factors are age, histology, stage, stromal invasion, tumor size, and tumor grade. The relationship between the ABO and Rh system with cervical cancer has been studied since the 1950s, though without obtaining clear results. Here we investigated the association between the ABO blood group and Rh system and consecutively treated cervical cancer patients in our department.

**Methods:** Clinical charts of cervical cancer patients treated and followed from 2010 to 2021 were checked for inclusion and exclusion criteria. Clinical and pathological data were recorded in a separate, anonymous, password-protected electronic database. All relevant data were extrapolated and used for final analysis.

**Results:** A population of 143 cervical cancer patients was analyzed in this study. 47.6% (68/143) were blood group O, 36.4% (52/143) were blood group A, 8.4% (12/143) were blood group AB, and 7.7% (11/143) were blood group B. 14.9% (21/141) were RhD negative, while 85.1% (120/141) were RhD positive. No significant association was found between the ABO group and survival. However, patients with blood types B and AB had a higher BMI than the other blood types. RhD-negative patients exhibited a lower age at diagnosis (P=0.035) and had a higher overall survival compared to RhD-positive patients.

**Conclusions:** The RhD factor appears to influence cervical cancer OS, but the data are too weakly significant to draw a definitive conclusion. Further studies with larger samples are needed to confirm this finding and to investigate the true impact of blood groups in female cancers.

## Introduction

Cervical cancer (CC) is the most common genital cancer and the fourth most frequent female cancer, with approximately 604,000 new cases and 342,000 deaths in 2020 worldwide 1. It is most frequently diagnosed in women with an average age at diagnosis of 50 2 and presents with diverse histological types. Squamous cell carcinoma (SCC) is the predominant histological variant, representing approximately three-fourths of all CC cases. Adenocarcinoma contributes to 10-15% of cases, while the remaining 10-15% are categorized under other or unspecified histologies 3. The main cause of CC is a persistent infection of the human papillomavirus (HPV), which can be detected in 99.7% of patients affected. It is a sexually transmitted infection, often contracted during early adulthood 4, but the introduction of the Pap smear in 1950 by George Papanicolaou is considered the paramount event that reduced CC 5. HPV persistence is also the principal factor associated with an increased risk of recurrence of high-grade cervical dysplasia after laser conization and the loop electrosurgical excision procedure 6,7. Nowadays, CC is considered a preventable disease, thanks to both effective screening strategies and vaccination against the most carcinogenic HPV strains 8. Several studies have shown that HPV vaccines are effective in preventing infection and precancerous lesions thanks to a cross efficacy against non-vaccine HPV types 9. Thus, HPV vaccination is offered free of charge in some countries after excisional treatment of a high-grade lesion to prevent recurrence. Moreover, there are also very rare histotypes unrelated to HPV such as gastric, mesonephric, neuroendocrine, carcinosarcoma, cervical adenoid carcinoma, primary lymphoma and melanoma of the cervix 10-12. CC treatment depends on many factors, including the histotype, stage, age, and the patient's desire for parenthood, and can vary from simple conization to radical hysterectomy surgery or chemo- and radiotherapy 13, 14. Well-known prognostic factors include age, histology, the International Federation of Gynecology and Obstetrics (FIGO) stage, deep stromal invasion, tumor size, tumor grade, metastasis, surgery, chemotherapy, radiation sequence with surgery and lymph node dissection 15. Since the 1950s, when the percentage of blood group A was found significantly higher among CC patients compared to the general population 16, the relationship between the ABO/Rh system and CC has been investigated, though without obtaining clear results (Table 1). ABO blood group antigens are complex carbohydrate molecules expressed in red blood cells and in other cell lines and tissues. Growing evidence highlights that ABO antigens, in addition to their key role in transfusion medicine, also interplay with the pathogenesis of many human disorders, including neoplastic diseases 17. Evidence of an association between ABO blood group antigens and various types of cancers has been investigated by several studies 17-23. The relationship between genetic variants of the ABO locus and the mechanism by which the ABO blood group is at interplay with cancer development and progression remains an open question. Alterations in the inflammatory state due to ABO blood group antigens provide a potential mechanism by which blood type may affect the progression and spread of malignancy. The inflammatory response plays crucial functions in various stages of tumor formation, and it also has an impact on immune surveillance and treatment response 24. Previous studies from our group evidenced the relationship between the ABO group and gynecological cancer, specifically in ovarian 12 and endometrial cancer 23. Nevertheless, there is scarce knowledge about the correlation between the oncological outcome of CC and the ABO blood group 25, 26.

In this study, we employed a retrospective analysis to investigate the relationship between the ABO/Rh blood group and the outcome of CC patients consecutively treated in our Gynecologic Department.

## Materials and Methods

### Patient characteristics

Our study was designed following the Strengthening the Reporting of Observational Studies in Epidemiology (STROBE) statement 27, and was approved by the Provincial Ethics Committee of Reggio Emilia (2017/0112372) on 11/27/2017.

Written informed consent was obtained from all participants to use personal non-sensitive data at hospital admission, in agreement with the guidelines on good clinical practice (DL 06/11/2007) and the European General Data Protection Regulation (EU GDPR 2016/679). The relevant ethics committee of the Area Vasta Emilia Nord approved the protocol of this study.

All patients with cervical cancer were treated and followed up at the AUSL-IRCCS of Reggio Emilia (Italy) from 2010 to 2021 and consequently treated in our department based on the ABO/Rh information available.

Patients with previous or concomitant cancers and patients with severe co-morbidities that posed an imminent risk to survival were excluded from the study. Instead, we included patients with the most common comorbidities such as hypertension, obesity, diabetes.

Clinical and pathological data were recorded in a separate, anonymous, password-protected electronic database. All relevant data were extrapolated and used for final analysis.

### Treatments

Treatment was planned based on the stage of CC and risk factors such as size of the tumour, stromal invasion, tumor differentiation, lymph-vascular space invasion (LVSI), status of resection margins, status of parametria and vaginal cuff, status of lymph nodes. Patients with FIGO stage IA1 without LVSI were treated with conization only if negative margins or with simple hysterectomy. In case of LVSI simple hysterectomy was associated with systematic pelvic lymph adenectomy (PLND) ± systematic para-aortic lymph adenectomy (PALND). Patients with FIGO stage IA1 CC underwent to radical or simple hysterectomy + PLND ± PALND. Patients with FIGO IB2 or IIA underwent to radical hysterectomy + PLND ± PALND. Patients with FIGO stage IB2 or IIB or IIIB underwent to chemoradiotherapy (CRT), and radiation was tailored according to surgical staging or positron emission tomography finding. Rarely neoadjuvant chemotherapy (CHT) followed by surgery or radiotherapy was performed. Patients with FIGO IVA underwent to CHT followed by radiotherapy and FIGO IVB patients underwent CHT only.

### Follow-up

Follow up visits were carried out in a multidisciplinary clinic by the gynecological oncologist and the radiotherapist and when necessary, by the medical oncologist. Patients with early-stage CC treated only with surgery underwent a complete physical examination with pelvic-rectal examination and transvaginal and abdominal ultrasound every six months for the first two years and every 12 months for the other three years. Cytology alone and in recent years co-tests to look for high-risk HPV positivity have been performed every 12 months. Abdominal and thoracic computed tomography and magnetic resonance imaging were performed two years after surgery and before the conclusion of follow up or in case of clinical suspicion of recurrence. In advanced-stage CCs undergoing multimodality treatment, the follow-up visit was performed every six months for five years, the medical oncologist was always present, and imaging examinations were performed every six months.

### Statistical analysis

The R statistical software package version 4.3.1 (R Foundation for Statistical Computing, Vienna, Austria) was used to perform statistical analysis. By applying Fisher's exact test and generalized linear models, we assessed univariate associations between ABO/Rh genotypes and clinical-pathological variables. Disease free survival (DFS) was calculated as the period spent from the treatment date to the date of first recurrence. The overall survival (OS) was calculated as the period spent from the treatment date to the date of death or last follow-up. For survival analysis, Kaplan-Meier curves were used to represent overall survival trends in the different groups of patients, and the log-rank test was applied to calculate p-value. Significant statements refer to p-values lower than 0.05.

## Results

### Patient characteristics

The study cohort included 143 women diagnosed with cervical cancer (Table 2). The evaluation was performed by analyzing the clinical and pathological characteristics of blood group/Rh factor, age at diagnosis, body mass index (BMI), parity, presence of diabetes mellitus (DM), histological types, surgical interventions, neoadjuvant and adjuvant treatments, cancer stage, recurrence rates, and OS.

The blood group distribution within enrolled women showed that 47.6% (68/143) were blood group O, 36.4% (52/143) were blood group A, 8.4% (12/143) were blood group AB, and 7.7% (11/143) were blood group B. Additionally, 14.9% (21/141) were RhD negative, while 85.1% (120/141) were RhD positive, with only two cases missing this classification.

The average age at the time of diagnosis was 54.6 years and the mean BMI was 24.7. Histological examinations identified adenocarcinoma in 30.3% (43/142) of cases, squamous cell carcinoma in 67.6% (96/142), and other histological types in 2.1% (3/142), with one case missing histology.

Within the cohort, 80.5% (107/133) of the women were parity, and the mean age of menopause (MP) was 46.4. Diabetes mellitus was present in 7.3% of cases. Hormone-related data revealed that 88.5% (108/122) of women did not use hormone replacement therapy (HRT), while 4.1% (5/122) used HRT, and 5.7% (7/122) used contraceptive pills. Additionally, 0.8% (1/122) underwent treatment with tamoxifen (TMX), and 0.8% (1/122) received progesterone therapy (PT) or the 0.8% (1/122) estrogen and progesterone (EP), with 21 patients missing information about HRT. Furthermore, 41.3% (59/143) of patients did not undergo surgery, while 58.7% (84/143) had a surgical intervention.

Cervical cancer staging revealed 47% (63/134) at stage I, 16.4% (22/134) at stage II, 21.6% (29/134) at stage III, and 14.2% (19/134) at stage IV. Neoadjuvant treatment was administered in 10.7% (15/140) of cases. Adjuvant treatment was given to 22.5% (32/142) of patients. Recurrence cases accounted for 26.2% (37/141), and the mortality rate was 30.1% (43/143). A comprehensive overview of the patients' clinical and pathological information is summarized in Table 2.

### ABO group and cervical cancer

Analysis of the association between ABO group and cervical cancer did not show any significant correlations. However, patients with blood group B and AB had a mean BMI of 28 and 28.5 respectively (overweight), while patients with blood groups O and A had normal weight on average, with mean BMI of 24.5 and 23.1, respectively (p-value=0.022) (Table 3).

**Figure 1:**
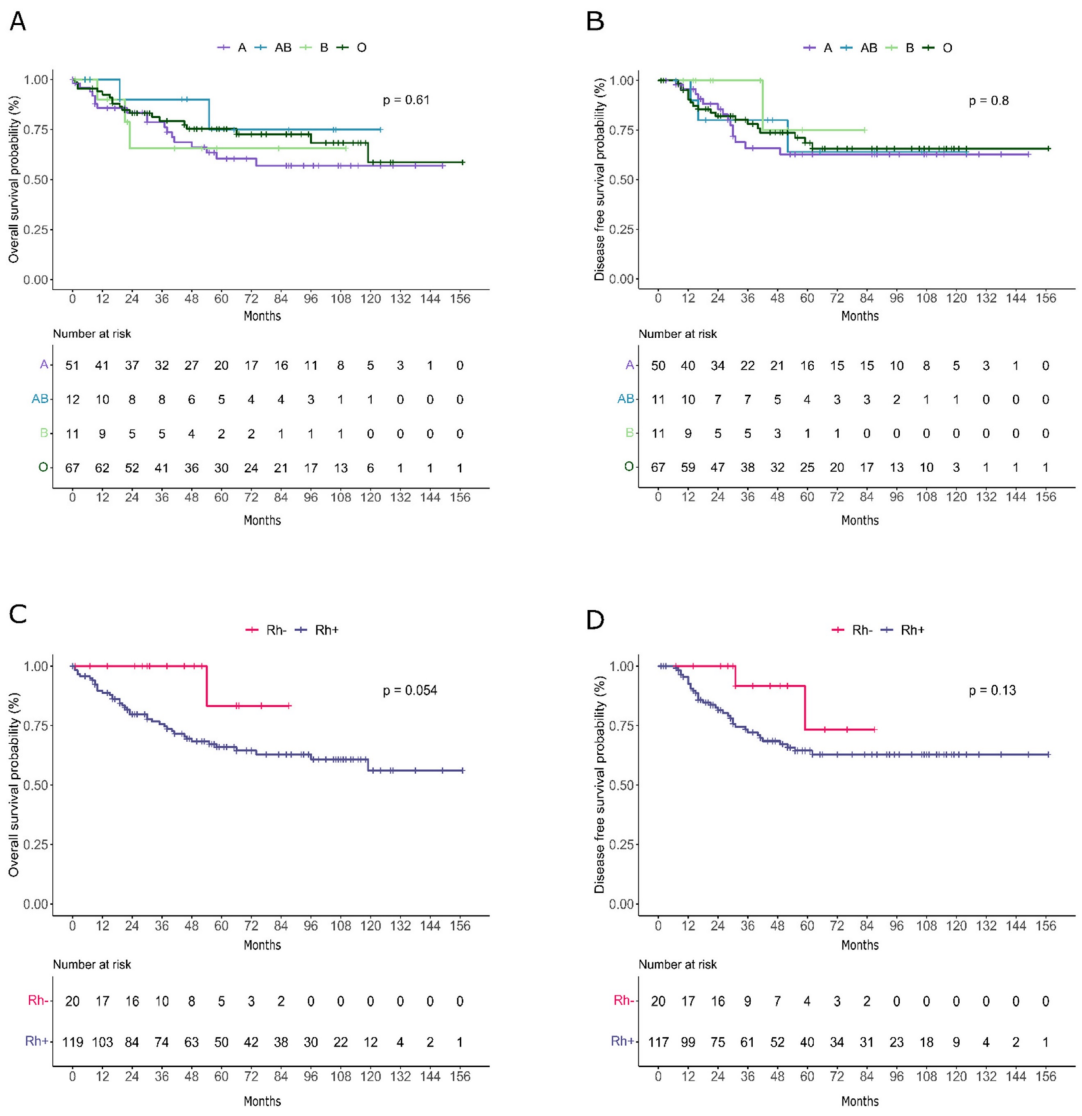
Kaplan-Meier curves representing overall survival (A-C) and disease free survival (B-D) trends in patients divided by AB0 (A-B) and RhD factor (C-D).

No significant association was seen between ABO group and OS (Figure 1A) and DFS (Figure 1B) in cervical cancer patients.

### Rh factor and cervical cancer

Analyses demonstrated a significant association between RhD and cervical cancer. As shown in Table 4, RhD-negative patients had a lower age at diagnosis (p=0.035) with a mean age of 48 years compared with a mean age of 55.8 years in patients with positive RhD, and a lower rate of hypertension (6.7% in RhD-negative vs 35.2% in RhD-positive, p=0.035). Interestingly, patients with negative RhD also showed a significantly lower rate of death (9.5% vs 34.2%, p=0.023). Remarkably, RhD-negative patients demonstrated higher OS compared to RhD-positive individuals, as depicted in the Kaplan-Meier curves (Figure 1C) and a similar trend was also observed for DFS (Figure 1D).

## Discussion

In our study, there was no correlation between ABO blood group and CC (Table 3). No impact of ABO blood group on CC survival was found (Figure 1 A-B). Conversely, a previous American Study showed that patients who underwent radiation therapy with blood group 0 had an approximately 20% higher 3-year survival rate than groups A and B combined. Furthermore, group B showed a trend towards a worse response than group A 28. Subsequently, a previous Italian study by Marinaccio et al. showed that a slightly better than 5-year survival was associated with the O blood phenotype; on the contrary, when a 10-year or longer survival was considered, a better survival was associated with the A blood phenotype 29. This reversal of impact on survival by the ABO group antigen could be due to the progressive loss of the CC cell surface isoantigen, with the consequent possible increase in the effectiveness of the immune system 29. A more recent study confirmed this finding by reporting that patients with early-stage CC with a blood type other than O have poorer recurrence-free survival compared to blood type O, which is evident during the first 5 years 25. These different results from our finding could be due to the different populations under study, the first coming specifically from a city in southern Italy 29 and the second from Thailand 25. No other studies regarding ABO blood groups and outcomes of CC patients have been reported in the literature. Most studies have investigated the frequency of ABO blood groups in CC patients or compared to the general population, showing different results (Table 1). Some studies have reported an increased frequency of blood group A in CC patients 16, 30-34. In the first study reported in the literature, group A was significantly more frequent in the CC patients (41.7%) than the other blood groups (B group: 19.6%, O group: 34.7% and AB group: 9.7%) 16. A study by Gupta found a 14% higher frequency of group A in CC patients compared to the general population 31. Similarly, Kaur et al. showed that blood group A women have a 15% greater chance of acquiring CC than blood group O women, whereas blood group B women have a 10% greater chance if compared to O blood group women 32. On the contrary, the study by Fotra et al. showed that the frequency of blood group B (41.53%) was the highest, followed by blood group O (21.77%), blood group AB (15.9%), and blood group A was the lowest (13.30%) 35. A similar finding was reported by Kai et al., where group B and early age of marriage were significantly associated with CC risk in the Indian population 36. In another study, the incidence of CC was higher in the AB group compared with the O group 37. According to our review, several studies from different countries (India, the United States, Iran, southeast Siberia, Italy, Denmark) found no correlation between ABO blood group and CC 26, 38-43. A recent retrospective cohort study on 291,680 women found positive associations of blood group A with both “mucous polyp of cervix” and blood group AB with “cervicitis and endocervicitis”, but there was no association with CC or cervical dysplasia 43. However, a meta-analysis of 6,029 CC patients found an ethnicity-specific association between A group and CC in Caucasian patients (OR = 1.09, 95% CI: 1.00-1.19) 17. Unlike most previous studies that investigated the effect of the RhD factor on the risk of developing CC and not on CC survival, we found a slight association between positive RhD and worse survival (Figure 1 C-D); the weakness of the result is probably due to the small sample size. However, no evidence to conclude that the RhD system is associated with CC has been reported in the previous studies found in the English language literature 26, 32, 35, 41, 43, 44. Although CC can be diagnosed as early as age 20 or in women in their 60s, it is usually diagnosed between ages 35 and 54. Interestingly, in our cohort, RhD-positive patients developed CC at a later age (mean age 56 years) than RhD-negative patients (mean age 48 years). The worse prognosis of RhD-positive patients might be related to their advanced age. According to previous studies, increasing age was linked to a detrimental effect on survival 45 probably because the patients underwent a less radical therapy. The comorbidities of elderly patients with CC can contribute substantially to the patient's prognosis, influencing their ability to receive and tolerate standard treatment 46.

The mechanism to explain how the ABO/Rh system can alter the immune response is still under investigation. Inflammation, immune surveillance for malignant cells, intercellular adhesion and membrane signaling could be involved in the interaction between ABO systems and cancers 47. Sugars and carbohydrates are glycans and are predominant components of the surface of cells such as erythrocytes. It is precisely through glycans that tumor cells can suppress the immune response, alter the microenvironment and increase angiogenesis, thus promoting tumor growth 48. Free glycans promote the proliferation and metastasis of cancer cells, and anti-glycan antibodies may neutralize free glycans and protect against cancer 49-52. ABO antigens may interfere with cell adhesion, cell signaling and immune surveillance 53, 54. ABO gene polymorphism has been implicated in susceptibility to several cancers across different populations 55. This gene encodes for glycosyltransferases, which catalyze the transfer of single sugars to the H antigen to form the A and B antigen 56, 57. Blood group O persons, who do not have the A and B gene coded glycosyltransferase, express a fucosylated variant of the precursor structure 58. The lack of expression of blood group antigens in tumors correlates with the absence of blood group-encoded glycosyltransferase 59. Aberrant glycosylation patterns are a hallmark of cancer development and progression 60, 61, and aberrant glycosylation occurs early during oncogenic transformation and may represent a key event in invasion and metastasis.

It is well known that in cancers such as endometrial, bladder and oral carcinoma, the loss of A and B antigens is correlated with the degree of malignancy and metastatic potential. 53, 60, 62. In CC, decreased expression of blood group antigens occurs early in cancer transformation; it seems to precede the cytological abnormalities used for the morphological diagnosis of dysplasia, and it is a progressive loss until cancer develops 63, 64. Some authors found that antigen expression was retained in two-thirds of the patients, regardless of stage, and that negative antigen expression was associated with a worse prognosis (5-year overall survival was 37% in antigen-negative group vs 71% antigen-positive group) 65. In another study, CC patients with an invasion depth greater than 1.5 cm and with negative antigen expression showed a poor prognosis compared to patients with positive antigen expression 66, 67. These findings therefore suggested using loss of blood group antigen expression as a useful predictive marker to select patients for adjuvant therapy.

Antigen-negative tumors have reduced levels of ABO transcript as compared to A antigen-positive tumors 60. The regulatory mechanism of ABO gene transcription presents two promoter regions 68, 69. Expression of the ABO gene in epithelial and erythroid cell lines has been shown to be dependent on the methylation status of the proximal constitutive promoter encoding most of the ABO transcripts, as an inverse relationship was found between promoter hypermethylation and ABO gene expression 68. Hence, poorly differentiated tumors contain high amounts of fully methylated alleles. The levels of DNA methylation have been shown to increase with the degree of malignancy. Hypermethylation in hyperplastic or dysplastic epithelium is found, and it may therefore be an early sign of malignant transformation 60. Fortunately, there are some future perspectives on the detection and prevention of cancer. In a recent study by Luo et al., experiments showed that tumors expressing ABO blood group antigens can be used as a new strategy for treating solid tumors 70. The blood group antigens bind to the corresponding antibodies in human serum to stimulate the body's immune system, induce erythrocyte-like lysis, eliminate tumor cells, and reduce tumor size. With the aim of lysing tumor cells and achieving the purpose of eliminating tumors, patients with blood type A might choose blood group B antigens for treatment, while patients with blood type B might choose blood group A antigens for treatment, thus activating the body's immune system and eliminating tumor cells. The outcomes of these experiments *in vivo* with animal models will be useful for the development of novel gene therapy approaches for solid tumor treatment. Regarding common comorbidities such as hypertension, we found that RhD-positive patients were associated with a higher risk of hypertension compared to RhD-negative patients (Table 4). Previously, Medalie et al. found that RhD-negative men had the lowest rate of development of hypertension (incidence 59% in 3427 RhD-positive men but only 29% in 311 RhD-negative men) 71. In a cohort of hypertensive patients of African ethnicity, of which 72.7% were women, the RhD factor was positive in 84.8% of cases 72. In a previous study on Rh blood group polymorphisms, the Rh genotype was significantly associated with systolic blood pressure (p=0.006) regardless of age, gender, weight, and BMI 73. The greater predisposition to hypertension in RhD-positive patients might also be linked to the increased risk of peripheral angiopathy (incidence rate ratios: 1.18 (1.05,1.31) 43. Our study presents some limitations due to the retrospective design with its potential biases and confounders. Moreover, it might be underpowered due to the small cohort studied and the findings could be limited to ethnicity. Nevertheless, the centralization of diagnosis, treatment, follow-up and ABO assessment guaranteed homogeneous management and reliable data. Furthermore, the characteristics of the CC patients included in our study were similar to those of other CC populations, confirming the reliability of the study cohort.

In conclusion, most previous studies have investigated the effect of blood type and RhD factor on the risk of developing CC and not their impact on CC survival, particularly of the RhD factor. Our study showed that a RhD-negative factor may influence CC OS, although the data are weakly statistically significant. Moreover, we confirm the association between RhD blood group and the risk of hypertension. We failed to find an association between ABO blood type and CC survival, but considering that ABO antigens are widely expressed on different human cells and perform different functions, it seems difficult to exclude their role in CC. In the future, ABO antigens could be an indicator of preneoplastic transformation and cancer progression as well as a useful economic prognostic factor for guiding adjuvant therapy.

## Funding

This study was partially supported by Italian Ministry of Health - Ricerca Corrente Annual Program 2025.

## Figures and Tables

**Table 1:** The ABO blood groups and RhD system in cervical cancer patients: results reported in literature

YEAR	FIRST AUTHOR (reference)	ETHNICITY	SAMPLE SIZE	AGE (n)	FIGO STAGE (n)	0 % (n)	*P* * value*	A % (n)	*P value*	B % (n)	*P value*	AB % (n)	*P value*	CONCLUSION
1957	Segi M et al. (16)	Japan	1534	-	-	29.1% (446) Cervical cancer	-	41.7% (60) Cervical cancer	0.01	19.8 (304) Cervical cancer	-	9.4% (144) Cervical cancer	-	The percentage of **group A cases was significantly higher among cancer** patients.
						30.5% (161461) Controls		38.3% (203255) Controls		21.8% (115416) Controls		9.4% (49914) Controls		
1962	Mitra S et al. (38)	India	521 Cervical Cancer	-	-	32.28% (172)		22.78% (114)		38.31% (204)		6.63% (31%)		There was **no significant relationship of ABO blood groups with cervical cancer.**
			2.273 Controls			32.60 % (740)		26.10% (594)		34.20% (777)		7.10% (162)		
1963	Garriga R et al. (28)	United States of America	123	-	I (67)	**Good** **response** 84.2% (48)	**A+B versus O:** **P>** **0.02** **-**	**Good** **response** 66.6% (28)	A versus 0: P= 0.1	**Good** **response** 63.3% (14)	B versus 0: *P=0.2*	Sample too small to perform analysis: 2/123 (1.6%)		**0 group had a higher 3-year survival rate by about 20%** than groups A and B combined. **B group exhibited** a trend toward **poorer response than group A**.
					II (54)	**Poor** **response** 15.8% (9)		**Poor** **response** 33.3% (14)		**Poor** **response** 36.4% (8)				
1965	Rotkin ID (44)	United States of America	185 Cervical Cancer	48.7 (range 22-79)	185 Cervical Cancer	50% (93) Rh^+^37.8% (70) Rh 5.9% (11)	<0.27	45% (84) Rh^+^ 30.8% (57) Rh^-^ 6.5% (12)	<0.42	16.8% (31) Rh^+^ 14% (26) Rh^-^ 2.7% (5)		2.2% (4) Rh^+^ 1.6% (3) Rh^-^ 0.5% (1)	<0.14	**No evidence** to conclude that blood groups and Rh+ are associated with cervical cancer.
			168 *In situ* Cervical Cancer		168 *in situ*	44% (74) Rh^+^37.5% (63) Rh 7.7% (13)	<0.32	41% (70) Rh^+^ 29.2% (49) Rh^-^ 6.5% (11)	<0.5	15.5% (26) Rh^+^ 13.1% (22) Rh^-^ 2.4% (4)		3.6% (6) Rh^+^ 3% (5) Rh^-^ 0.6% (1)	<0.4	
									
1967	Tyagi S. P. et al. (37)	India	556 Cervical Cancer	- 20-50	-	26.98% (150)	-	21.76% (121)	-	9.93% (222)	-	11.33% (63)	-	Relative incidence for cervical cancer in AB group was 1.82 as compared to incidence 1 in group 0 females.
			3022 Controls			31.96% (996)		22.01% (665) A:0 (RI 1.17 NS)		38.65% (1168) B:0 (RI 1.23 NS)		7.38% (223) AB:0 (RI1.82 S)		
1967	Janus ZL et al. (30)	United States of America	REVIEW											Cervical cancer patients tended to have a higher proportion of group A, because there is the possibility of bias in all series studied and because the relationship is small, we suspect there is no causal relationship between blood groups and cervical cancer.
1968	Gupta P. (31)	India	208 Cervical Cancer			23.56% (49)		37.02% (77)	**χ²=16.574** **p>0.01**	33.17% (69)		6.25% (13)		The patients suffering from cervical cancer show a strikingly high incidence in blood group A. and the difference is highly statistically significant.
			371 Controls			34.77% (129) -11.21%		22.10% (82) +14.92%		35.58% (132) -2.41%		7.55% (28) -1.3%		
1970	Mittal V. P. (39)	India	700 Cervical Cancer			32.43% (227)	NS	23.29% (163)	NS	36.71% (257)	NS	7.57% (53)	NS	No statistically significant difference in the A, B, O and AB blood group distribution.
			2000 Controls			28% (560) +4.43%		24.2% (484) +0.91%		38.5% (770) -1.79%		9.3% (186) -1.73%		
1974	Newell GR (40)	United States of America	745 Cervical Cancer			White females 42.7% (73)		44.4% (76) RR 1.27		7.6% (13)		5.3% (9)		Probably no causal relationship between blood groups and cervical cancer.
						Black females 50% (287)		23.7% (136) RR 0.96		22.8% (131)		3.5% (20)		
			550 Controls			White females 52.8% (89)		39.7% (77)		9.3% (18)		5.2% (10)		
						Black females 49.2% (175)		24.4% (87) RR 0.96		21.3% (76)		5.1% (18)		
1987	Marinaccio (42)	Italy	1043 Cervical Cancer			48.7%	NS	32.1%	NS	15.3%	NS	3.9%	NS	Distribution of ABO phenotypes among carcinoma of the uterine cervix is almost comparable to that of the healthy population.
			44327 Controls			50.3%		32.3%		13%		4.4%		
1992	Kaur I. et al. (32)	India	186 Cervical Cancer	- 35-60y		20.97% (39)		40.86% (76)		27.42% (51)		10.75% (20)		Woman with blood group A have a 15% greater probability of acquiring carcinoma of cervix uteri than group O women; women with blood group B have a 10% greater probability than group O women.
			274 Controls			26.28% (72)		32.48 (89) A:0 RI 1.5765		34.67% (95) B:0 RI 0.9911		6.75% (18)		
Rh^+^ Rh^-^ CC 88.71% (165) 11.29% (21) C 92.7% (254) 7.3% (20)														There is no significant association with the RhD system.
1995	Marinaccio M et al. (29)	Italy	639		I (186) 5ys 10ys	81.7% 67/82 47.6% 39/82	**0/A** **0.007** **0/A 0.0001** **0/A 0.0001** **0/A 0.006**	72.9% (43/59) 50.8% (28/39)		71.8% (3/39) 7.7% (5/6)		83.3% (3/6) 50% -		A slightly better than 5-year survival is associated with O blood phenotype; on the contrary, when a 10 year or longer survival is considered, a better survival is associated with A blood phenotype.
					II (338) 5ys 10ys	57.8% (96/166) 22.3% (37/166)		44.3% (51/115) 31.3% (36/115)		37.8% (17/45) 8.8% (4/45)		58.3% (7/12) 33.3% (4/12)		
					I+II	65.2% (163/248) 30.7% (76/248)		54% (94/174) 37.9% (66/174)		53.6% (45/84) 8.3% (7/84)		66.6% (12/18) 38.9% (7/18)		
					III (115) 5ys 10ys	18.2% (10/55) 16.4% (9/55)		14.3% (6/42) 4.8% (2/42)		6.7% (1/15) 6.7% (1/15)		33.3% (1/3) 33.3% (1/3)		
					GM 5ys 10ys	57.1% (173/303) 28% (85/303)		46.3% 100/216 31.5% 68/216		46.5% (44/99) 8.1% (8/99)		61.9% (13/21) 38.1% (8/21)		
2011	Fotra R. et al. (35)	India	248 Cervical carcinoma			21.77% (54)		13.3% (39)		41.53% (103)		15.9% (33)		B blood group has the strongest association with cervical cancer.
			254 Controls			26% (66)		21.7% (55)		42.5% (108)		9.8% (25)		
Rh^+^ Rh^-^ CC92.3% (229) 7.66% (19) C 96.9% (246) 3.14% (8)														
2012	Yuzhalin A.E. et al. (41)	Russia	172 Cervical cancer	30-75y -		36.1% (62)	-	36% (62)	0.9122	23.3% (40)	0.9308	4.7% (8)	0.1027	No statistically significant correlations were found for cervical cancer. Additionally, no relationship was observed between Rhesus factor and cancer risk.
			22581 Control group			35% (6570)		34.4% (6570)		22.2% (4248)		8.4% (1599)		
Rh^+^ Rh^-^ CC83.1% (143) 17.4% (96) C 84.3% (19032) 15.7% (3549)														
2013	Kai L. J. et al. (36)	India	100 Cervical cancer	-	-	29% (29)		12% (12)		55% (55)	**<0.001**	4% (4)		Blood group B and age of marriage between 11 and 20 years were significantly associated with cervical cancer.
			200 Controls			39% (78)		17% (34)		31% (62) χ2 test = 18.2		13% (26)		
2016	Hanprasertpong J et al. (25)	Thailand	413 Cervical cancer	47 Mean age	32 IA2 381 IB1	39.23 % (162)	0.14	24.46 % (101)	0.01	30.75 % (127)		AB 5.57 % (23)		The ABO blood group was not associated with patient age, histology, LVSI, DSI, PI, node status, surgi- cal margin, or adjuvant therapy. However, the study found that pa- tients with blood type A had a higher percentage of FIGO stage IA2 (P= 0.010). Furthermore, patients with blood type O had a higher percentage of smaller lesions (P= 0.014).
						7.4% (12)		14.8% (15)		3.2% (4)		4.3% (1)		
						92.6% (150)		85.2% (86)		96.8% (123)		95.7% (22)		
						Tumor size <2cm 76.5% (124)		<2cm 63.4% (64)		<2cm 59.8% (76)		<2cm 60.9% (146)		
						>2cm 23.5% (38)		>2cm 36.6% (37)		>2cm 40.2% (51)		>2cm 39.1% (9)		
2021	Abegaz S. B. (33)	Ethiopia	REVIEW											Blood type A people have a greater occurrence of cervical cancer (13%), as compared to blood type O people.
2023	Joudaki N. et al. (26)	Iran	14 Cervical cancer	45.85 Mean age (range 13-74)		57.2%		7.1%		7.1%		28.6%	p 0.421	No relevance between ABO and Rh blood groups and breast and cervix cancer.
			29,922 Controls			40%		7.0%		25%		28%		
Cervical cancer (%) Rh^+^ 92.9% Rh^ -^ 7.1% p-value 1.000 Healthy population (%) 92.38% 7.62%														
2023	Cui H. et al. (17)	China	METANALISIS 6029 cases 1658278 Controls (15 datasets)					**A vs O** OR (95%CI) 1.17 (1.02-1.35) P effect 0.025		**B vs O** OR (95%CI) 1.13 (1.03-1.23) P effect 0.011		**AB vs O** OR (95%CI) 1.01 (0.83-1.23) P effect 0.928		A group was significantly associated with risk of (…) cervical cancer.
2023	Bruun-Rasmussen P. et al. (43)	Denmark	Cervical cancer and dysplasia 12538			IRR (95% CI) 1 (0.99, 1.01)	0.97	IRR (95% CI) 1.05 (0.99, 1.12)	0.118	IRR (95% CI) 0.9 (0.83, 0.97)	**0.01**	IRR (95% CI) 0.96 (0.66, 1.39)		Positive associations of blood group B with both cervical cancer and dysplasia.
			Cervical intraepithelial neoplasia [CIN] 10895			1 (0.84, 1.19)	0.97	1.06 (0.99, 1.13)	0.115	0.89 (0.82, 0.97)	**0.009**	0.95(0.67, 1.34)		

RhD-positive blood group relative to the RhD negative blood group Cervical cancer and dysplasia IRR (95% CI) 0.93 (0.86, 1.01) p-value 0.083 Cervical intraepithelial neoplasia [CIN] IRR (95% CI) 0.93 (0.85, 1.01) p-value 0.102

**Table 2:** Summary of patients' clinical and pathological data

	Overall (N=143)
**Age**	
Mean (SD)	54.6 (15.5)
**Parity**	
No	26 (19.5%)
Yes	107 (80.5%)
N-Miss	10
**Body Mass Index**	
Mean (SD)	24.7 (5.8)
N-Miss	47
**Diabetes Mellitus**	
No	115 (92.7%)
Yes	9 (7.3%)
N-Miss	19
**Hypertension**	
No	84 (68.9%)
Yes	38 (31.1%)
N-Miss	21
**Hormone replacement therapy**	
No	108 (88.5%)
Yes	5 (4.1%)
Contraceptive pills	7 (5.7%)
Tamoxifen	1 (0.8%)
Progesterone/Estrogen therapy	1 (0.8%)
N-Miss	21
**AB0**	
A	52 (36.4%)
AB	12 (8.4%)
B	11 (7.7%)
0	68 (47.6%)
**RH**	
Negative	21 (14.9%)
Positive	120 (85.1%)
N-Miss	2
**Symptoms**	
Pap smear	8 (5.6%)
No	66 (46.2%)
Occasional finding	3 (2.1%)
Yes	66 (46.2%)
**Histotype**	
Adenocarcinoma	43 (30.3%)
Squamous cell carcinoma	96 (67.6%)
Other	3 (2.1%)
N-Miss	1
**Surgery**	
No	59 (41.3%)
Yes	84 (58.7%)
N-Miss	0
**Neoadjuvant therapy**	
No	125 (89.3%)
Yes	15 (10.7%)
N-Miss	3
**Adjuvant therapy**	
No	110 (77.5%)
Yes	32 (22.5%)
N-Miss	1
**Stage**	
I	63 (47.4%)
II	22 (16.5%)
III	29 (21.8%)
IV	19 (14.3%)
N-Miss	10
**Recurrence**	
No	104 (73.8%)
Yes	37 (26.2%)
N-Miss	2
**Death**	
No	100 (69.9%)
Yes	43 (30.1%)
**Follow up**	
Mean (SD)	56.9 (40.9)

**Table 3:** Association between AB0 group and cervical cancer

	O (N=68)	A (N=52)	B (N=11)	AB (N=12)	Total (N=143)	p value
**Age**						0.295
Mean (SD)	55.7 (16.0)	55.0 (15.9)	46.1 (14.0)	54.5 (9.5)	54.6 (15.5)	
**Body Mass Index**						0.022
Mean (SD)	24.5 (5.4)	23.1 (5.2)	28.0 (9.3)	28.5 (3.6)	24.7 (5.8)	
N-Miss	23	19	2	3	47	
**Diabete Mellitus**						0.553
No	54 (90.0%)	41 (95.3%)	9 (90.0%)	11 (100.0%)	115 (92.7%)	
Yes	6 (10.0%)	2 (4.7%)	1 (10.0%)	0 (0.0%)	9 (7.3%)	
N-Miss	8	9	1	1	19	
**Ipertension**						0.834
No	42 (68.9%)	28 (68.3%)	8 (80.0%)	6 (60.0%)	84 (68.9%)	
Yes	19 (31.1%)	13 (31.7%)	2 (20.0%)	4 (40.0%)	38 (31.1%)	
N-Miss	7	11	1	2	21	
**RH factor**						0.769
Negative	8 (11.9%)	9 (17.6%)	2 (18.2%)	2 (16.7%)	21 (14.9%)	
Positive	59 (88.1%)	42 (82.4%)	9 (81.8%)	10 (83.3%)	120 (85.1%)	
N-Miss	1	1	0	0	2	
**Histotype**						0.649
Adenocarcinoma	25 (37.3%)	12 (23.1%)	3 (27.3%)	3 (25.0%)	43 (30.3%)	
Squamous cells carcinoma	41 (61.2%)	38 (73.1%)	8 (72.7%)	9 (75.0%)	96 (67.6%)	
Other	1 (1.5%)	2 (3.8%)	0 (0.0%)	0 (0.0%)	3 (2.1%)	
N-Miss	1	0	0	0	1	
**Surgery**						0.979
No	29 (42.6%)	21 (40.4%)	4 (36.4%)	5 (41.7%)	59 (41.3%)	
Yes	39 (57.4%)	31 (59.6%)	7 (63.6%)	7 (58.3%)	84 (58.7%)	
N-Miss	0	0	0	0	0	
**Neoadjuvant therapy**						0.625
No	60 (90.9%)	46 (90.2%)	9 (81.8%)	10 (83.3%)	125 (89.3%)	
Yes	6 (9.1%)	5 (9.8%)	2 (18.2%)	2 (16.7%)	15 (10.7%)	
N-Miss	2	1	0	0	3	
**Adjuvant therapy**						0.846
No	51 (75.0%)	41 (80.4%)	8 (72.7%)	10 (83.3%)	110 (77.5%)	
Yes	17 (25.0%)	10 (19.6%)	3 (27.3%)	2 (16.7%)	32 (22.5%)	
N-Miss	0	1	0	0	1	
**Stage**						0.970
I	28 (43.1%)	24 (51.1%)	6 (60.0%)	5 (45.5%)	63 (47.4%)	
II	12 (18.5%)	6 (12.8%)	2 (20.0%)	2 (18.2%)	22 (16.5%)	
III	16 (24.6%)	9 (19.1%)	1 (10.0%)	3 (27.3%)	29 (21.8%)	
IV	9 (13.8%)	8 (17.0%)	1 (10.0%)	1 (9.1%)	19 (14.3%)	
N-Miss	3	5	1	1	10	
**Stage (grouped)**						0.689
I	28 (43.1%)	24 (51.1%)	6 (60.0%)	5 (45.5%)	63 (47.4%)	
II-III-IV	37 (56.9%)	23 (48.9%)	4 (40.0%)	6 (54.5%)	70 (52.6%)	
N-Miss	3	5	1	1	10	
**Recurrence**						0.673
No	49 (72.1%)	37 (72.5%)	10 (90.9%)	8 (72.7%)	104 (73.8%)	
Yes	19 (27.9%)	14 (27.5%)	1 (9.1%)	3 (27.3%)	37 (26.2%)	
N-Miss	0	1	0	1	2	
**Death**						0.537
No	49 (72.1%)	33 (63.5%)	8 (72.7%)	10 (83.3%)	100 (69.9%)	
Yes	19 (27.9%)	19 (36.5%)	3 (27.3%)	2 (16.7%)	43 (30.1%)	

**Table 4:** Association between RhD and cervical cancer

	NEGATIVE (N=21)	POSITIVE (N=120)	Total (N=141)	p value
**Age**				0.035
Mean (SD)	48.0 (13.8)	55.8 (15.6)	54.6 (15.6)	
**Body Mass Index**				0.416
Mean (SD)	23.545 (5.904)	24.976 (5.858)	24.778 (5.853)	
N-Miss	8	39	47	
**Diabete Mellitus**				0.599
No	15 (100.0%)	98 (91.6%)	113 (92.6%)	
Yes	0 (0.0%)	9 (8.4%)	9 (7.4%)	
N-Miss	6	13	19	
**Hypertension**				0.035
No	14 (93.3%)	68 (64.8%)	82 (68.3%)	
Yes	1 (6.7%)	37 (35.2%)	38 (31.7%)	
N-Miss	6	15	21	
**AB0**				0.753
A	9 (42.9%)	42 (35.0%)	51 (36.2%)	
AB	2 (9.5%)	10 (8.3%)	12 (8.5%)	
B	2 (9.5%)	9 (7.5%)	11 (7.8%)	
O	8 (38.1%)	59 (49.2%)	67 (47.5%)	
N-Miss	0	0	0	
**Histotype**				0.312
Adenocarcinoma	9 (42.9%)	33 (27.7%)	42 (30.0%)	
Squamous cells carcinoma	12 (57.1%)	83 (69.7%)	95 (67.9%)	
Other	0 (0.0%)	3 (2.5%)	3 (2.1%)	
N-Miss	0	1	1	
**Surgery**				0.096
No	5 (23.8%)	53 (44.2%)	58 (41.1%)	
Yes	16 (76.2%)	67 (55.8%)	83 (58.9%)	
N-Miss	0	0	0	
**Neoadjuvant therapy**				1.000
No	19 (90.5%)	104 (88.9%)	123 (89.1%)	
Yes	2 (9.5%)	13 (11.1%)	15 (10.9%)	
N-Miss	0	3	3	
**Adjuvant therapy**				0.783
No	17 (81.0%)	91 (76.5%)	108 (77.1%)	
Yes	4 (19.0%)	28 (23.5%)	32 (22.9%)	
N-Miss	0	1	1	
**Stage**				0.133
I	12 (57.1%)	50 (45.5%)	62 (47.3%)	
II	5 (23.8%)	17 (15.5%)	22 (16.8%)	
III	4 (19.0%)	24 (21.8%)	28 (21.4%)	
IV	0 (0.0%)	19 (17.3%)	19 (14.5%)	
N-Miss	0	10	10	
**Stage (grouped)**				0.350
I	12 (57.1%)	50 (45.5%)	62 (47.3%)	
II-III-IV	9 (42.9%)	60 (54.5%)	69 (52.7%)	
N-Miss	0	10	10	
**Recurrence**				0.063
No	19 (90.5%)	83 (70.3%)	102 (73.4%)	
Yes	2 (9.5%)	35 (29.7%)	37 (26.6%)	
N-Miss	0	2	2	
**Death**				0.023
No	19 (90.5%)	79 (65.8%)	98 (69.5%)	
Yes	2 (9.5%)	41 (34.2%)	43 (30.5%)	

## Abbreviations

CC: cervical cancer
SCC: squamous cell carcinoma
HPV: human papillomavirus
FIGO: International Federation of Gynecology and Obstetrics
STROBE: Strengthening the Reporting of Observational Studies in Epidemiology
EU GDPR: European general data protection regulation
LVSI: lymph-vascular space invasion
PLND: systematic pelvic lymph adenectomy
PALND: systematic para-aortic lymph adenectomy
CRT: chemoradiotherapy
CHT: chemotherapy
DFS: disease free survival
OS: overall survival
HR: hazard ratio
BMI: body mass index
DM: diabetes mellitus
MP: menopause
HRT: hormone replacement therapy
TMX: tamoxifen
TP: progesterone therapy
EP: estrogen and progesterone therapy

## References

[1] Buskwofie A, David-West G, Clare CA (2020). A Review of Cervical Cancer: Incidence and Disparities. J Natl Med Assoc.

[2] Johnson CA, James D, Marzan A (2019). Cervical Cancer: An Overview of Pathophysiology and Management. Semin Oncol Nurs.

[3] Vinh-Hung V, Bourgain C, Vlastos G (2007). Prognostic value of histopathology and trends in cervical cancer: a SEER population study. BMC Cancer.

[4] Fontham ETH, Wolf AMD, Church TR (2020). Cervical cancer screening for individuals at average risk: 2020 guideline update from the American Cancer Society. CA Cancer J Clin.

[5] Eun TJ, Perkins RB (2020). Screening for Cervical Cancer. Med Clin North Am.

[6] Bogani G, Sopracordevole F, Di Donato V (2021). High-risk HPV-positive and -negative high-grade cervical dysplasia: Analysis of 5-year outcomes. Gynecol Oncol.

[7] Bogani G, DI Donato V, Sopracordevole F (2020). Recurrence rate after loop electrosurgical excision procedure (LEEP) and laser Conization: A 5-year follow-up study. Gynecol Oncol.

[8] Shin HY, Lee B, Hwang SH (2019). Evaluation of satisfaction with three different cervical cancer screening modalities: clinician-collected Pap test vs HPV test by self-sampling vs HPV test by urine sampling. J Gynecol Oncol.

[9] Canfell K, Kim JJ, Brisson M (2020). Mortality impact of achieving WHO cervical cancer elimination targets: a comparative modelling analysis in 78 low-income and lower-middle-income countries. Lancet.

[10] Mandato VD, Palermo R, Falbo A (2014). Primary diffuse large B-cell lymphoma of the uterus: case report and review. Anticancer Res.

[11] Bifulco G, Mandato VD, Giampaolino P (2009). Small cell neuroendocrine cervical carcinoma with 1-year follow-up: case report and review. Anticancer Res.

[12] Mandato VD, Kobal B, Di Stefano A (2009). Amelanotic malignant melanoma of the uterine cervix with ten-year follow-up. Eur J Gynaecol Oncol.

[13] Bogani G, Donato VD, Scambia G (2022). Practice patterns and 90-day treatment-related morbidity in early-stage cervical cancer. Gynecol Oncol.

[14] Cibula D, Raspollini MR, Planchamp F (2023). ESGO/ESTRO/ESP Guidelines for the management of patients with cervical cancer - Update 2023. Int J Gynecol Cancer.

[15] Hu C, Cao J, Zeng L (2022). Prognostic factors for squamous cervical carcinoma identified by competing-risks analysis: A study based on the SEER database. Medicine (Baltimore).

[16] Segi M, Fujisaku S, Kurihara M (1957). Cancer of cervix uteri and ABO blood groups. Tohoku J Exp Med.

[17] Cui H, Qu Y, Zhang L (2023). Epidemiological and genetic evidence for the relationship between ABO blood group and human cancer. Int J Cancer.

[19] Bezek T, Bingulac-Popović J, Bagatin D (2022). Abo blood group genotypes in women with breast cancer. Acta Clin Croat.

[20] Hamada T, Oyama H, Nakai Y (2021). ABO Blood Group and Risk of Pancreatic Carcinogenesis in Intraductal Papillary Mucinous Neoplasms. Cancer Epidemiol Biomarkers Prev.

[21] Machlowska J, Baj J, Sitarz M (2020). Gastric Cancer: Epidemiology, Risk Factors, Classification, Genomic Characteristics and Treatment Strategies. Int J Mol Sci.

[22] Mandato VD, Torricelli F, Mastrofilippo V (2019). AB0 Blood Group and Ovarian Cancer Survival. J Cancer.

[23] Mandato VD, Torricelli F, Mastrofilippo V (2017). Prognostic Impact of ABO Blood Group on Type I Endometrial Cancer Patients- Results from Our Own and Other Studies. J Cancer.

[24] Grivennikov SI, Greten FR, Karin M (2010). Immunity, inflammation, and cancer. Cell.

[25] Hanprasertpong J, Jiamset I, Atjimakul T (2016). Prognostic value of ABO blood group in patients with early stage cervical cancer treated with radical hysterectomy with pelvic node dissection. Tumour Biol.

[26] Joudaki N, Khodadadi A, Talaiezadeh A (2023). Study of the Relationship between ABO Blood Group Types and Breast Cancer and Cervix Cancer in Khuzestan Province, Iran. Int J Hematol Oncol Stem Cell Res.

[27] Vandenbroucke JP, von Elm E, Altman DG (2007). Strengthening the Reporting of Observational Studies in Epidemiology (STROBE): explanation and elaboration. PLoS Med.

[28] Garriga R, Ghossein NA (1963). The ABO blood groups and their relation to the radiation response in carcinoma of the cervix. Cancer.

[29] Marinaccio M, Traversa A, Carioggia E (1995). Gruppi sanguigni del sistema ABO e sopravvivenza nei tumori ginecologici [Blood groups of the ABO system and survival rate in gynecologic tumors]. Minerva Ginecol.

[30] Janus ZL, Bailar JC 3rd, Eisenberg H (1967). Blood group and uterine cancer. Am J Epidemiol.

[31] Gupta P (1968). ABO blood groups and their relationship with cancer of the cervix uteri. J Indian Med Assoc.

[32] Kaur I, Singh IP, Bhasin MK (1992). Blood groups in relation to carcinoma of cervix uteri. Hum Hered.

[33] Abegaz SB (2021). Human ABO Blood Groups and Their Associations with Different Diseases. Biomed Res Int.

[34] Gualandri V, Cozzi M (1967). Sui rapporti fra i gruppi sanguigni del sistema ABO e le neoplasie dell'apparato genitale femminile. II. Cancro dell'utero [On the relations between blood groups of the ABO system and neoplasms of the female genital system. II. Cancer of the uterus]. Minerva Ginecol.

[35] Fotra R, Upma U, Gupta S (2011). Association of ABO and Rh Blood Groups With the Carcinoma of the Cervix With Special Reference to Jammu Region. Biosci Biotech Res Asia.

[36] Kai LJ, Raju K, Malligere Lingaiah HK (2013). Significance of blood group and social factors in carcinoma cervix in a semi-urban population in India. Asian Pac J Cancer Prev.

[37] Tyagi SP, Tiagi GK, Pradhan S (1967). ABO blood groups in relation to cancer cervix. Indian J Med Sci.

[38] Mitra S, Mondal S, Basu A (1962). The study of ABO blood groups in cancer of the female genital organs and cancer of the breast. Cancer.

[39] Mittal VP (1970). Blood groups and cancer of the cervix uteri. J Obstet Gynaecol India.

[40] Newell GR, Gordon JE, Monlezun AP (1974). ABO blood groups and cancer. J Natl Cancer Inst.

[41] Yuzhalin AE, Kutikhin AG (2012). ABO and Rh blood groups in relation to ovarian, endometrial and cervical cancer risk among the population of South-East Siberia. Asian Pac J Cancer Prev.

[42] Marinaccio M, Trerotoli C (1987). Sistema ABO e neoplasie ginecologiche. Folia Oncol.

[43] Bruun-Rasmussen P, Hanefeld Dziegiel M, Banasik K (2023). Associations of ABO and Rhesus D blood groups with phenome-wide disease incidence: A 41-year retrospective cohort study of 482,914 patients. Elife.

[44] Rotkin ID (1965). Are ABO and Rh blood groups associated with cancer of the uterine cervix?. Cancer.

[45] Quinn BA, Deng X, Colton A (2019). Increasing age predicts poor cervical cancer prognosis with subsequent effect on treatment and overall survival. Brachytherapy.

[46] Barben J, Kamga AM, Dabakuyo-Yonli TS (2022). Cervical cancer in older women: Does age matter?. Maturitas.

[47] Wolpin BM, Chan AT, Hartge P (2009). ABO blood group and the risk of pancreatic cancer. J Natl Cancer Inst.

[48] Purohit S, Ferris DG, Alvarez M (2020). Better survival is observed in cervical cancer patients positive for specific anti-glycan antibodies and receiving brachytherapy. Gynecol Oncol.

[49] Gates MA, Wolpin BM, Cramer DW (2011). ABO blood group and incidence of epithelial ovarian cancer. Int J Cancer.

[50] Li Y, Liu L, Huang Y (2020). Association of ABO polymorphisms and pancreatic Cancer/ Cardiocerebrovascular disease: a meta-analysis. BMC Med Genet.

[51] Campbell CT, Gulley JL, Oyelaran O (2013). Serum antibodies to blood group A predict survival on PROSTVAC-VF. Clin Cancer Res.

[52] Carvalho HA, Villar RC (2018). Radiotherapy and immune response: the systemic effects of a local treatment. Clinics (Sao Paulo).

[53] Hakomori S (1999). Antigen structure and genetic basis of histo-blood groups A, B and O: their changes associated with human cancer. Biochim Biophys Acta.

[54] Melzer D, Perry JR, Hernandez D (2008). A genome-wide association study identifies protein quantitative trait loci (pQTLs). PLoS Genet.

[55] Duan YF, Zhu F, Li XD (2015). Association between ABO gene polymorphism (rs505922) and cancer risk: a meta-analysis. Tumour Biol.

[56] Yazer MH (2005). What a difference 2 nucleotides make: a short review of ABO genetics. Transfus Med Rev.

[57] Watkins WM (1966). Blood-group substances. Science.

[58] Dabelsteen E (2002). ABO blood group antigens in oral mucosa. What is new?. J Oral Pathol Med.

[59] Mandel U, Langkilde NC, Orntoft TF (1992). Expression of histo-bloodgroup-A/B-gene-defined glycosyltransferases in normal and malignant epithelia: correlation with A/B-carbohydrate expression. Int J Cancer.

[60] Gao S, Worm J, Guldberg P (2004). Genetic and epigenetic alterations of the blood group ABO gene in oral squamous cell carcinoma. Int J Cancer.

[61] Hakomori S (2002). Glycosylation defining cancer malignancy: new wine in an old bottle. Proc Natl Acad Sci U S A.

[62] Kuemmel A, Single K, Bittinger F (2007). The prognostic impact of blood group-related antigen Lewis Y and the ABH blood groups in resected non-small cell lung cancer. Tumour Biol.

[63] Himes TR, Ernst CS, Koprowska I (1986). Loss of blood isoantigens in exfoliated cells during the progression of CIN demonstrated by monoclonal antibody staining. Acta Cytol.

[64] Moro-Rodríguez E, Alvarez-Fernández E (2008). Losses of expression of the antigens A, Lea and Lex and over-expression of Ley in carcinomas and HG-SIL of the uterine cervix. Diagn Pathol.

[65] Lindgren A, Stendahl U, Brodin T (1986). Blood group antigen expression and prognosis in squamous cell carcinoma of the uterine cervix. Anticancer Res.

[66] To AC, Soong SJ, Shingleton HM (1986). Immunohistochemistry of the blood group A,B,H isoantigens and Oxford Ca antigen as prognostic markers for stage IB squamous cell carcinoma of the cervix. Cancer.

[67] Ichikawa D, Handa K, Withers DA (1997). Histo-blood group A/B versus H status of human carcinoma cells as correlated with haptotactic cell motility: approach with A and B gene transfection. Cancer Res.

[68] Kominato Y, Hata Y, Takizawa H (1999). Expression of human histo-blood group ABO genes is dependent upon DNA methylation of the promoter region. J Biol Chem.

[69] Kominato Y, Hata Y, Takizawa H (2002). Alternative promoter identified between a hypermethylated upstream region of repetitive elements and a CpG island in human ABO histo-blood group genes. J Biol Chem.

[70] Luo Q, Pan M, Feng H, Wang L (2021). ABO blood group antigen therapy: a potential new strategy against solid tumors. Sci Rep.

[71] Medalie JH, Papier C, Goldbourt U (1973). Blood groups and hypertension. Isr J Med Sci.

[72] Sacomboio ENM, Sassoke JL, Hungulo OFS (2021). Frequency of AB0/Rh Blood Groups and Social Condition of Hypertensive Patients in Luanda. J Blood Disord Med.

[73] Robinson MT, Wilson TW, Nicholson GA (2004). AGT and RH blood group polymorphisms affect blood pressure and lipids in Afro-Caribbeans. J Hum Hypertens.

